# Ruptured Pulmonary Hydatid Cyst Following the Use of Albendazole

**DOI:** 10.4269/ajtmh.17-0915

**Published:** 2018-06

**Authors:** Rahul Daimari, Anthony A. Oyekunle, Cassandra Ocampo

**Affiliations:** Department of Internal Medicine, University of Botswana, Gaborone, Botswana

**Figure 1.**
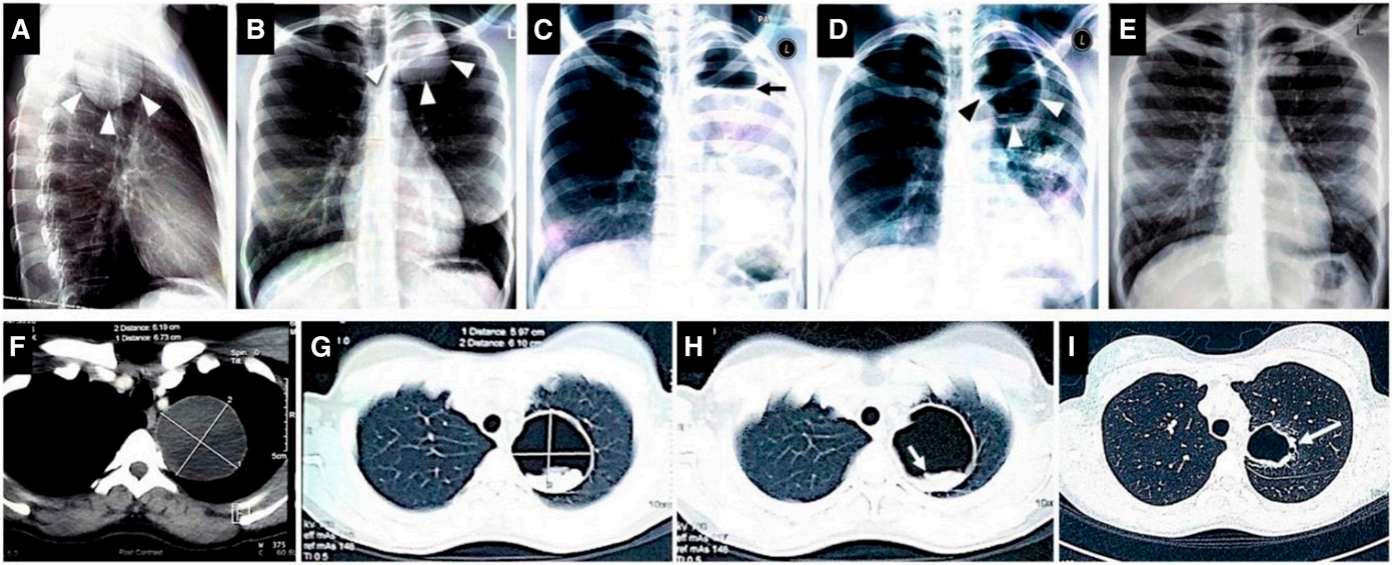
Plain radiograph (**A**–**E**) and computed tomographic (**F**–**I**) images of a 23-year-old female patient with pulmonary hydatid cyst disease. They show the large intact cyst at diagnosis (**A**, **B**, and **F**), left pulmonary interstitial involvement following rupture of the cyst (**C**), the cyst containing dependent residual tissue believed to be from the ruptured cyst wall (**G** and **H**), the cyst communicating with a bronchiole (**I**), and sequential evidence of recovery of pulmonary tissue (**D** and **E**). This figure appears in color at www.ajtmh.org.

A 23-year-old HIV-negative Botswana native was found to have had a sizeable pulmonary opacity (Figure 1A and B). High-resolution CT scan revealed a large (6.19 × 6.73 cm) solitary homogenous cystic lesion in the left upper lobe (Figure 1F), with anterior lobulation but no calcification. A therapeutic trial with albendazole was started. Three months into therapy, she reported with a 1-week history of severe shortness of breath and fever, with associated cough, productive of yellowish, foul-smelling, and salty sputum. She never kept pets and denied eating pork, sheep meat, or unwashed vegetables.

Clinical examination revealed tachycardia and tachypnea, with the use of accessory respiratory muscles. Breath sounds were decreased in the left upper zone. Laboratory investigations showed leukocytosis, with neutrophilia (ANC 13.2 × 10^3^/µL). A repeat radiograph (Figure 1C) showed the previously noted cyst with an air–fluid level (black arrow) and extensive diffuse opacification of the left lung suggestive of parenchymal involvement. Contrast chest CT (Figure 1G–I) confirmed rupture, with an intra-cyst membrane (“water lily” sign), and bronchiolar communication (Figure 1I). An echinococcus hemagglutination test was positive. She received antibiotics for pneumonia and restarted albendazole, with clinical improvement, as confirmed in chest radiographs taken 2 weeks post-rupture (Figure 1D). Cardiothoracic opinion on admission was in favor of conservative management. Four months after rupture, she remained asymptomatic and clinically stable, with chest radiograph that showed almost total resolution (Figure 1E).

Larvae of the tapeworm *Echinococcus* spp. cause hydatid disease (echinococcosis) in humans and most commonly occur in the liver and lungs. Among the four species, *Echinococcus granulosus*, *Echinococcus multilocularis*, *Echinococcus vogeli*, and *Echinococcus oligarthrus*, *E. granulosus* is found worldwide.^1^ Echinococcosis has been reported from Botswana but it is not a “high-endemic” area.^2^ The adult tapeworm resides in the canine intestines (definitive host) from where gravid proglottids or parasite eggs are shed in feces. The intermediate hosts (sheep, goats, and humans) are infected by ingesting these eggs. The cyst grows gradually, and patients may remain asymptomatic for years. Most lung cysts resolve by fibrosis and disappear within 5–9 months of treatment.^3^ Viable hydatid pulmonary cysts enlarge at 1–5 cm/year until they rupture or are removed. Giant (> 10 cm size) cysts are rare^4^ and require surgical intervention. Most experts prefer cystotomy and closure of the bronchial opening with modified capitonnage.^5^ Within resource-limited settings, a conservative approach with pharmacotherapy may be warranted, but this needs close monitoring for possible complications such as a rupture.
